# Towards Multiple Model Synchronization with Comprehensive Systems

**DOI:** 10.1007/978-3-030-45234-6_17

**Published:** 2020-03-13

**Authors:** Patrick Stünkel, Harald König, Yngve Lamo, Adrian Rutle

**Affiliations:** 8 University of Paderborn, Paderborn, Germany; 9 ICREA, Open University of Catalonia, Barcelona, Spain; 10 Høgskulen på Vestlandet, Bergen, Norway; 11 University of Applied Sciences, FHDW, Hannover, Germany

**Keywords:** Model Synchronization, Multimodelling, Multidirectional Transformations (MX), Inter-Model Consistency, Model Merging, Graph Diagrams, Triple Graph Grammars, Category Theory

## Abstract

Model management is a central activity in Software Engineering. The most challenging aspect of model management is to keep models consistent with each other while they evolve. As a consequence, there has been increasing activity in this area, which has produced a number of approaches to address this synchronization challenge. The majority of these approaches, however, is limited to a binary setting; i.e. the synchronization of exactly two models with each other. A recent Dagstuhl seminar on multidirectional transformations made it clear that there is a need for further investigations in the domain of general multiple model synchronization simply because not every multiary consistency relation can be factored into binary ones. However, with the help of an auxiliary artifact, which provides a global view over all models, multiary synchronization can be achieved by existing binary model synchronization means. In this paper, we propose a novel *comprehensive system* construction to produce such an artifact using the same underlying base modelling language as the one used to define the models. Our approach is based on the definition of partial commonalities among a set of aligned models. Comprehensive systems can be shown to generalize the underlying categories of graph diagrams and triple graph grammars and can efficiently be implemented in existing tools.

### 1 Introduction

Conceptual *models*, i.e. abstract specifications of the system under development, are recognized to be of major importance in software engineering [52]. Representing the whole system in a single global model is generally unfeasible, hence, different teams design and maintain several models which focus on different aspects of the system. This collection of inter-related models is often referred to as a *multimodel*. A rigorous use of these models within the engineering process eventually requires consistency management of multimodels. This is because the collection of models must obey global consistency rules and as models are inevitably subject to change, global consistency becomes an issue [16].

*Model Synchronization* represents a means to maintain global consistency of inter-related models by combining consistency verification with (semi-)automatic consistency restoration. The cross-disciplinary research field *Bidirectional Transformations (BX)* [8] investigates such means within different communities and it provides a number of theoretical and practical results (see [2] for a recent survey). However, the majority of these approaches is limited to a binary setting, i.e. keeping pairs of models consistent. Stevens [44] recognized this limitation in her outreach to the modelling community that lead to an increased momentum in this area as evident from a recent Dagstuhl seminar on *Multidirectional Transformations (MX)* [7].

One way to address multiary synchronization is to consider it as a network of well-understood binary synchronization problems. However, not every multiary consistency rule can be factored into binary ones [9]; e.g. the class diagrams $A^1$, $A^2$ and $A^3$ in fig. 1 are pairwise consistent but not altogether—since class inheritance is acyclic. Thus, multiary model synchronization is needed to keep global consistency. Another approach to global consistency management is the *model merge* approach [6]: It constructs the union of all models wherein the related elements are identified, see lower half of fig. 1 (inter-relations given by sameness of class' names). Thus, global consistency can be verified within a single artifact, the merge. However, the major drawback of this approach, apart from requiring additional computational overhead, is that it forgets the origin of elements; e.g. that class `C` was contained in $A^1$ and $A^2$ but not in $A^3$. This is a problem if global consistency rules depend on this containment information.

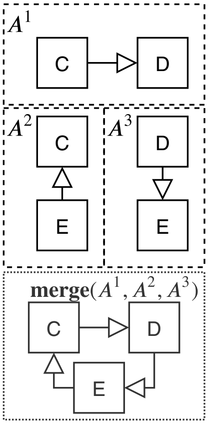

**Fig. 1.** Inconsistent class diagrams

The most important information in multiary model synchronization are the inter-relations between models and their elements. We call the latter *commonalities* and cannot generally assume that they are always given by equality of names as it was the case in fig. 1. Thus, multimodels must be extended with such commonality information, which allows element traceability and global consistency verification. Aligning models via an additional commonality structure has some tradition, e.g. it is the foundation of *Triple Graph Grammars (TGGs)* [40], a formal and mature BX approach with a focus on Model Driven Engineering (MDE). In the TGG approach, models are considered to have a *graph based* structure, i.e. there is a common underlying *base modelling language* and we will also stick to this idea of a common base language.

In this paper, we propose a novel construction called *comprehensive system* which serves as a foundation for various ways of multiary model management. It is based on a simple, non-intrusive and easy-to-handle *linguistic extension* of the base modelling language with commonality specifications, which allows to work with an arbitrary number $n \geq 2$ of heterogeneously typed (*local*) models as one single (*global*) model. Moreover, we will show that we are still able to apply mature methods for model verification and restoration in the same way as for single local models. Furthermore, we show that this approach is more expressive than, and overcomes the obstacles of, the model merge approach, and that it generalizes TGGs and *graph diagrams* [48] – a recent generalization of TGGs.

Before defining comprehensive systems and their properties (sect. 5 and 6), we clarify terminology (sect. 2), introduce of a running example (sect. 3), and provide an overview of the state of the art (sect. 4). An extended version of the proofs in sect. 6 is given in the technical report [47].

### 2 Preliminaries: Multimodelling

Every fast moving research field is prone to produce separate terms for the same concepts. Thus, we begin with a short definition of the most important terms in multi-model consistency management. We will stick to the imperative of MDE [42] and consider all Software Engineering (SE) artifacts as models:

**Model** A model is an abstract specification of the system (or parts of it) under development. Models are atomic elements in the multimodel consistency management process. To be amenable for electronic processing, we assume them to be formal, i.e. following the format of a specific *modelling language*. We denote models by capital letters $A, A', A^1, A^2$ etc.

**Metamodel and Conformance** Every modelling language is specified by an artifact called *metamodel*. We denote metamodels by capital letters $M, M', M^1$, $M^2$ etc. Models must conform to their respective metamodel, i.e. the model must be well-structured w.r.t. the metamodel **and** fulfill all *constraints* imposed on the metamodel, thus further narrowing admissible model structure. The model is then called an *instance* of the metamodel. Conformance is also called *local* or *intra-model consistency*. We denote a single constraint by lowercase $\phi$ and a set of constraints by uppercase $\Phi$. A metamodel with a set of constraints $\Phi$ imposed on it will be written $M_\Phi$.

**Correspondence** is a relation among a set of models. It is a consequence of *commonalities* (common concepts) shared by these models. A collection of models together with a correspondence among them is called a *multimodel*. In the similar way as for local models, global *consistency rules* can be imposed on a multimodel. It is considered (*globally*) *consistent*, if all local constraints and global consistency rules are fulfilled. Consistency of a multimodel is also referred to as *inter-model consistency*.

**Model Space** A model space is a set of models together with changes among them. In an MDE setting it can be considered to be given by a metamodel $M$: The set of all instances of $M$ together with $M$-respecting instance changes, which describe how an instance $A'$ is the result of edits on $A$. We write $\mathbf{Mod}(M_\Phi)$ to denote the respective model space.

### 3 Use Case

We depict a *collaborative modelling* example within *healthcare*. More concretely, the task is to develop ICT support for a *patient referral* process. A referral is "the act of sending a patient to another physician for ongoing management of a specific problem with the expectation that the patient will continue seeing the original physician for co-ordination of total care" [41]. It is an important and recurring process in the healthcare domain. Hence, ICT-support is desirable [51].

At the same time, development remains tricky since it requires multiple actors (software vendors, government officials, hospitals and physicians) to agree on common data structures, processes and interfaces. For our example, let us assume that the design of the system follows a model-based approach and there are three different models, each covering a different aspect of the system: There is a *process* model $A^1$ denoted in *Business Process Model and Notation (BPMN)* [30], a *data* model $A^2$ denoted as a *Unified Modelling Language (UML) class diagram* [32], and a *decision* model $A^3$ denoted in *Decision Model and Notation (DMN)* [33].

These three models are depicted in fig. 2 (ignore the cyan lines for the moment). The central ingredient is the process model $A^1$. It represents a simplified version of the process developed in [51]. The process is triggered by a patient's appeal beginning with an introductory consultation. Afterwards the main part of the process begins: Information about the patient and its medical history is extracted while in parallel a consultant is selected via a `business-rule` activity. The patient information is then sent to the consultant. The consultant can either approve the referral or reject it. In the latter case, another consultant has to be found. If a consultant accepts the referral, the process is finished.

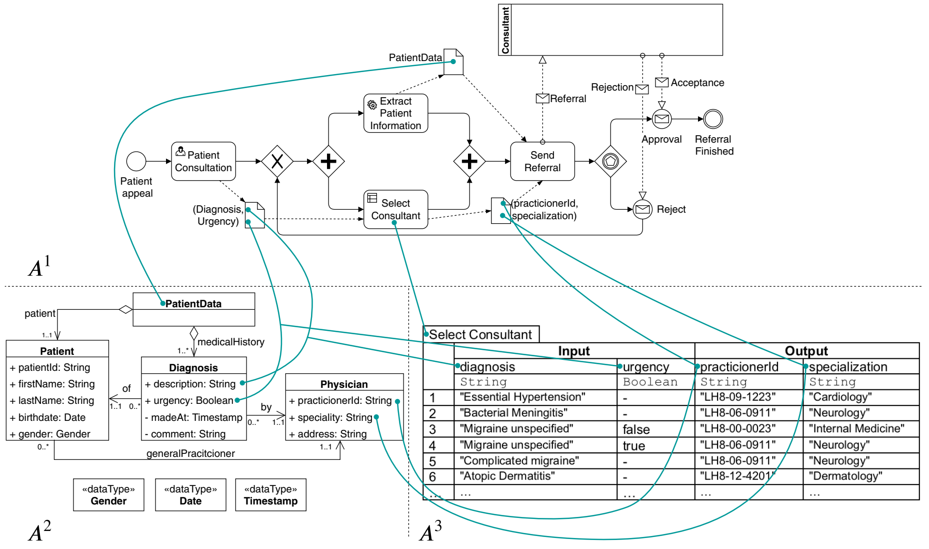

**Fig. 2.** Example models $A^1$, $A^2$ and $A^3$ and their commonalities

The other models in fig. 2 contain the respective data types ($A^2$) and specify the domain-specific behaviour of the “Select Consultant” activity ($A^3$). The latter is depicted as a table that assigns, for a given combination of values in input side columns, a combination of values in output side columns, i.e. based on `diagnosis` and `urgency`, an appropriate consultant is selected (which is identified by a `practicionerId` and `specialization`).

All models could be edited completely independent of each other would there not be a correspondence between them. It arises from the existence of abstractly “the same” information simultaneously contained in multiple models. Consider e.g. the column called `diagnosis` in $A^3$, which is reflected by a process variable in $A^1$ (visualized by a file symbol) and an attribute named `description` in $A^2$. We call these relations *commonalities* and depict them via cyan lines in fig. 2.

But the arising multimodel (models $A^1$, $A^2$, $A^3$ plus their commonalities) underlies *consistency rules* [11] (see sect. 2) which define consistency of a multimodel. For our example, assume the following consistency rules:

CR1 For every `business-rule` activity in $A^1$, there must exist a corresponding decision `table` in $A^3$ and vice versa.
CR2 Every `column type` in $A^3$ must refer to an existing `data type` in $A^2$ with the same `name`.
CR3 Every `column` in $A^3$ must have a corresponding `public attribute` (denoted by `+`) in $A^2$ and should be reflected by a `process variable` in $A^1$.
CR4 Every `process variable` in $A^1$ must either be reflected by a `class` or an `attribute` in $A^2$.

To actually maintain consistency of $A^1$, $A^2$ and $A^3$, w.r.t. CR1-CR4, we begin by a review of the state of the art how commonalities are identified, consistency is verified and if needed restored.

## 4 State of the Art

A seminal exposition of the process of *multimodel consistency management* is already given in [43]. It comprises four phases: (i) Detection of overlaps (we call them commonalities, see sect. 3, (ii) Detection of inconsistencies, (iii) Diagnosis of inconsistencies, and (iv) Handling of inconsistencies. The first step is also called *model alignment*. Many approaches do not consider an explicit diagnosis stage and combine (iii) and (iv) into a phase called *consistency restoration* a.k.a. *model repair* [28]. Hence, existing work can be grouped into these three categories:

**Alignment** The goal of model alignment is to identify relations between models, i.e. finding their commonalities. This procedure, a.k.a. *model matching*, has been studied in several domains: databases [35], ontologies [15], MDE [23], graph transformation [14] and software product lines [53]. Automatic model matching, in general, is NP-hard [36]. However, there may be domain-specific heuristics [53] which exploit underlying global identification mechanisms, e.g. social security numbers for persons or the ICD-10 ontology [54] for diseases. Surveys on this topic can be found in [15] (focus on ontologies), [35] (focus on databases) and [23] (focus on MDE). Further, it is important to note that model element matching requires that elements are transferable between models. This is e.g. directly given within the UML or multi-viewpoint modelling as there is a *single underlying metamodel* [3]. If this is not given a priori, matching on the level of metamodels [38,10] has to preceed the matching of model elements.

**Verification** The goal of consistency verification is to find all consistency violations. A recent survey on this topic is found in [22]. The focus of the authors is on UML but the results are universal. They present four categories to classify **v**erification approaches: ***s**ystem **m**odel (SMV)*, ***u**niversal **l**ogic (ULV)*, ***h**eterogeneous **t**ransformation (HTV)* and ***d**ynamic **m**etamodelling (DMV)*. In the SMV approach every model is translated into a comprehensive artifact where the verification is executed. ULV is a variant of the former where the translation is executed on the level of an underlying logic. HTV define translations between each pair of models and DMV considers extensions of each metamodel with elements from other metamodels or models to express global consistency.

**Restoration** A comprehensive survey about model repair approaches is found in [28], whereas [2] is a recent survey about BX based approaches. Insights from these surveys show that there are basically three categories of consistency **r**estoration approaches: ***p**rogramming **b**ased (PBR)* approaches where consistency and its restoration is explicitly defined simultaneously, ***s**olver **b**ased (SBR)* approaches where consistency is abstractly posed as logic formula and restoration is implemented using a solver or search-based algorithm, and finally, ***g**rammar **b**ased (GBR)* approaches such as TGGs [19], which place themselves somewhere in between. The big majority of these approaches, however, considers binary synchronization only. There are only few notable exceptions, e.g. the solver based *Echo* [29] and the *graph diagram* framework [48,49].

**Architecture** Analyzing the underlying system architecture of these approaches, there are, in principal, two designs: We call them the *network design* and the *span design*. Consider the multimodel as a graph where nodes represent models and edges represent correspondences (for alignment), consistency relations (for verification) or repair functions (for restoration). In the network design there are edges between each pair of models. In the span design the graph has a hub-and-spoke layout, i.e. there is an *additional* hub-node that has an edge towards every model. Approaches in the categories SMV, ULV and SBR are associated with a span design since they perform a translation into a an intermediate model, while approaches in the categories HTV, DMV and PBR are associated with the network design because they directly act on a pair of models. GBR approaches have used either of them.

Comparing the architecture, the network design puts the complexity on the edges whereas the span design puts complexity on the nodes (more specifically on a single node: the hub). The drawback of the network design is that the number of edges grows quadratically with the number of participating models and if consistency relations cannot be factored into binary relations, hyperedges are required, which further increase the complexity. Another issue with this design is the coordination of concurrent changes. The drawback of the span design is the additional overhead of the hub-node model, however, the hub-node provides a means to coordinate concurrent changes.

### 5 Comprehensive Systems

In this section, we introduce *comprehensive systems* (sect. 5.1 to 5.3), which follow a SMV-approach and mitigate the drawbacks of the span design. We will show in sect. 5.4 that comprehensive systems are a foundation for the PBR restoration approach and we conjecture that the same is true for SBR, because they do not fundamentally differ from the structure of local models, such that they can be fed into existing means for model verification and restoration. Moreover, sect. 5.5 shortly reports why our approach eliminates the model merge obstacles (see the discussion in the introduction and fig. 1).

Before introducing comprehensive systems concretely, we want to illustrate where they occur in typical conceptual workflows for multimodel consistency management. Fig. 3 depicts such a workflow which is more or less informally used in many approaches of multimodel management, e.g. [16]. It comprises the phases mentioned in sect. 4: alignment, verification and restoration. The result of the first stage are the comprehensive metamodel and global consistency rules imposed upon it, and metamodel element commonalities, which are stored persistently to avoid expensive re-computation and possible information loss, cf. motivation in [25]. These commonalities are then used to *compute* the comprehensive system under consideration, e.g. a model merge. It can be used in the subsequent phases shown in fig. 3.

In contrast to this *additional* computation, our definition of comprehensive system is based on a non-intrusive extension of existing models by commonalities *without extensive computations.* Furthermore, it enables natural internalizations of inter-relations between different local models into a single artifact. Our intention is to demonstrate this internalization informally in this section and formalize it in sect. 6, where we will also state that the resulting structure generalizes triple graphs [40] and graph diagrams [48]; hence it is ready to be used in GBR approaches, too.

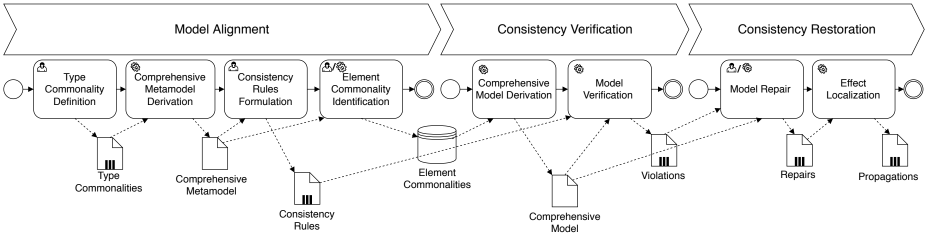

**Fig. 3.** General Multimodel Consistency Management Process

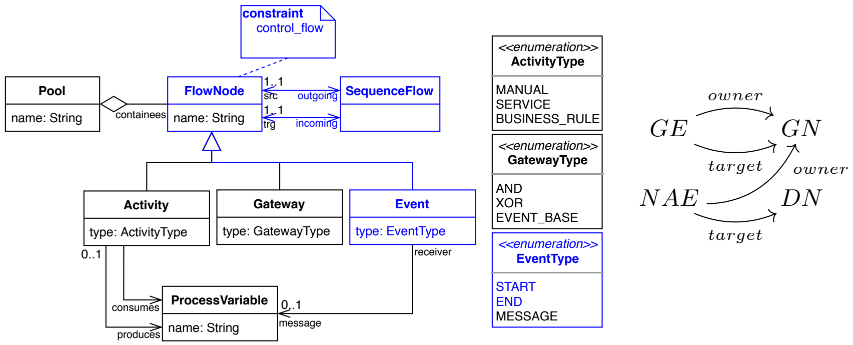

(a) BPMN metamodel $M^1$

(b) Simplified E-graph Signature

**Fig. 4.** Metamodel Example and Base Language

#### 5.1 Typed Local Models

We begin on the level of metamodels: Fig. 4a depicts a simplified metamodel $M^1$ of BPMN for our example. We do not endorse any specific MDE-framework and denote metamodels in a UML class diagram-like style. Metamodels $M^2$ and $M^3$ for UML class diagram and DMN models can be defined in the same way as metamodel $M^1$ (excerpts of them are shown in fig. 5). E-graphs [12] (see fig. 4b) give a formal interpretation to the class diagram syntax, which may serve as an appropriate *base modelling language* $\mathbb{B}$ for our purposes, i.e. a shared *linguistic (meta-)metamodel* [26]. It consists of Graph Nodes $GN$ and Data Nodes $DN$ (complex and primitive types in the UML terminology), as well as Graph Edges $GE$ (associations) and Node Attribute Edges $NAE$ (attributes) together with appropriate owner and target functions. For the sake of simplicity we omitted edge attribute edges, which are usually included in E-graphs. Every model $A$ must conform to a metamodel $M$. Since models and metamodels can be depicted as E-Graphs, the conformance relation is a *typing* homomorphisms $t : A \to M$ between the E-Graphs $A$ and $M$. If, e.g. $a$ is a flow node in $A^1$, see fig. 2, then $t(a) = \texttt{FlowNode} \in M^1$. Hence, model space $\mathbf{Mod}(M)$ is the category of E-graphs typed over $M$. E-graphs are only one possible base language and we will work with arbitrary base languages in sect. 6. Nevertheless will we use the term "*graph*" to subsume all artifacts under consideration (models and metamodels). Thus, we will use the terms (graph- and data-) "*nodes*" and (graph- and node attribute-) "*edges*" for the contents of these graphs, see [12] for the original terminology.

If a set $\Phi$ of *constraints* (e.g. a set of formulas given in a specific logic) is imposed on $M$, then the space is reduced to the full subcategory $\mathbf{Mod}(M_\Phi)$ of all consistent models typed over $M$ w.r.t. $\Phi$. Besides *UML-internal constraints* (e.g. the `1..1`-multiplicity on `src` and `tgt` in fig. 4a) given in the modelling technique, there are often *attached constraints* $\phi \in \Phi$. An example for an attached constraint is $\phi :=$`control_flow`, see the note at `FlowNode` in fig. 4a. This constraint defines that every `Start Event` must not have any incoming `SequenceFlow` [30, p. 237], whereas an `End Event` must not have any outgoing `SequenceFlow` [30, p. 245]. Listing 1.1 shows an *Object Constraint Language (OCL)* [31] formulation of this constraint.

**Listing 1.1.** Constraint $\phi$:=`control_flow` formulated in OCL

```
context FlowNode inv:
   self.oclIsTypeOf(Event) and self.eventType=EventType::START) implies
      self.incoming->count() = 0
   and (self.oclIsTypeOf(Event) and self.eventType=EventType::END) implies
      self.outgoing->count() = 0
```

OCL is just an example of a possible means for defining attached constraints. As we do not endorse a specific metamodelling framework and thus also not endorse a specific technique for the definition of attached constraints, we treat all constraints uniformly and assume that all internal and external constraints can be modelled as *diagrammatic constraints* [37]. A diagrammatic constraint $\phi$ imposed on a metamodel $M$ possesses an "arity graph" $S_\phi$ and is imposed on $M$ by a scope $d_\phi : S_\phi \to M$ (a homomorphism). The semantics is provided by a predicate $check_\phi : \mathbf{Mod}(S_\phi) \to Bool$, which verifies whether a given structure typed over the arity fulfills this constraint. The scope highlights a fragment (the image of $d$) of metamodel $M$, e.g. the blue coloured fragment in fig. 4a is the scope of the constraint $\phi$ from listing 1.1. For a typed graph $t : A \to M$, the verification procedure $verify(t) = check_\phi(query(t))$ comprises two steps: First, *query* forgets all elements of $A$ not typed over the scope, then it retypes the remaining elements w.r.t. $d$ such that they are typed over $S_\phi$. That is, *query* implements the *pullback* of $d$ and $t$. Finally, $check_\phi$ is invoked on the pullback result.

#### 5.2 Extending the Base Language

As seen in sect. 3, consistency rules play a major role in multimodelling. However, we cannot directly formalize them via the diagrammatic constraints described above since their definition involves elements spanning multiple models. Note that inter-relations between models arise from models sharing abstractly the "same" real-world concepts (see the intuitive cyan lines in fig. 2). We name these structural relations *commonalities* and they are also well-known in practice as traceability links [16,39,1]. There are different interpretations of what such a link can mean, e.g. identity, subset, extension? etc. [16]. In our framework commonality semantics are kept abstract, i.e. considering them as any kind of structural relation allowing us to define diagrammatic constraints in multimodels.

For example, in order to formalize CR2, we need to declare a commonality between the terms `DataType` (in $M^2$) and `ColumnType` in $M^3$. In addition to these *binary* commonalities in which only two terms are matched, there are also ternary commonalities, e.g. `String` occurs in all three metamodels and it is necessary to relate BPMN-term `ProcessVariable` with UML-term `Attribute` and DMN-term `Column` together with their respective `name`- and `type`-features to express CR3. These declarations may be formulated in an intuitive domain-specific language (DSL) shown in listing 1.2.

**Listing 1.2.** Type Commonalities

```
1  commonalities (BPMN,UML,DMN) {
2   relate(BPMN.String,UML.String,DMN.String) as String;
3   relate(BPMN.Activity,DMN.Table) as Decision;
4   relate(BPMN.ProcessVariable,UML.Attribute,DMN.Column)
5    as Var with {
6     relate(BPMN.name,UML.name,DMN.name) as name;
7     relate(DMN.type,UML.type) as type; };
8   relate(UML.DataType,DMN.ColumnType) as Type
9    with { relate{UML.name,DMN.name} as name; };
10  relate(BPMN.ProcessVariable,UML.Class) as Entity; }
```

The specification in listing 1.2 *extends* the modelling artifacts $M^1, M^2$ and $M^3$ and we call its syntax a *linguistic extension*. Each `relate`-statement translates to an object, which is identified by an alias (keyword `as`) and which reifies the "tupling" of terms it relates. E.g. the object `Var` in lines 4-7 specifies a commonality of the triple `ProcessVariable` ($M^1$), `Attribute` ($M^2$), and `Column` ($M^3$). `Var` is an object in its own right and we call it a *(commonality) representative.*

However, not only the nodes (of the graphs) should be related: In listing 1.2 we see that the keyword `with` defines the two features, i.e. edges, `type` and `name` of the respective graphs to be related as well. Common edges require that their respective source and target nodes are also related, e.g. the `type`-commonality entails commonality of `Attribute` and `Column`, which is already given by the surrounding `relate`-statement, as well as commonality of `DataType` and `ColumnType` (see lines 8-9). Hence, commonality specifications must preserve edge-node-incidences.

Consequently, it is reasonable to use the same language $\mathbb{B}$ for commonality representatives. In such a way, a commonality specification is itself an E-graph: The semantic interpretation of listing 1.2 is depicted in cyan in fig. 5. The proper linguistic extension further comprises mappings, which assign to each commonality representative $w$ the elements it relates. E.g. `Decision` is mapped to `Activity` and to `Table` in the respective metamodels. Since the assignment syntax in the above DSL also contains the target metamodel of the related elements (e.g. *`BPMN`* in `relate(BPMN.Activity...)`), these mappings decompose into 3 *projection mappings* $p_j : M^0 \to M^j$ $(j \in \{1,2,3\})$, depicted by dotted arrows in fig. 5, e.g. $p_1$(`Decision`) $=$ `Activity` $\in M^1$, as well as $p_2$(`Type`) $=$ `DataType` $\in M^2$, the target metamodel now encoded in $p$'s index. Since the corresponding tuples can be of arbitrary arity, these mappings may be partial:

$$p_1(w') = \perp, p_2(w') = \texttt{DataType}, p_3(w') = \texttt{ColumnType}$$

if $w' =$ `Type`. Finally, the above required edge-node-incidence means that definedness of $p_j(e)$ entails definedness of $p_j(v)$, where $v$ is the source of $e$, and

$$p_j(v) = \text{ source of } p_j(e) \tag{1}$$

for all edges $e$ in $M^0$ (and likewise for targets).

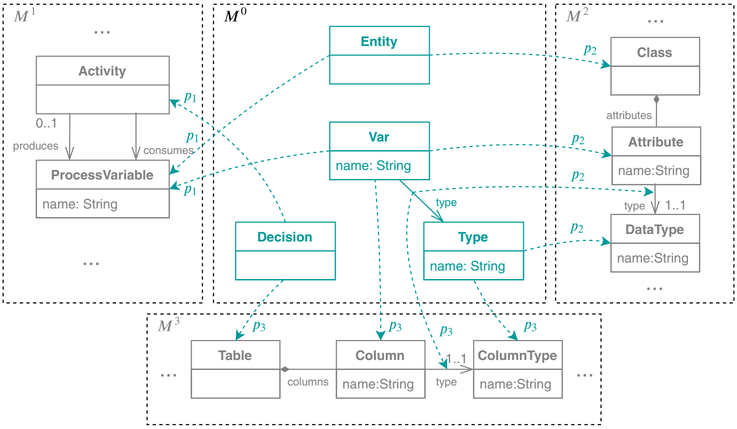

**Fig. 5.** Commonality representative metamodel $M^0$

#### 5.3 Metamodel and Model Commonalities

The previous section showed that a linguistic extension of the base language with projection functions between commonality representatives and the elements they relate yields an alignment of metamodels $M^1, \ldots, M^n$. The result is a comprehensive metamodel, in which commonalities are accurately specified with the help of (a graph of) commonality representatives. Formally, we obtain a new graph $M^0$ and partial projections

$$M^0 \xrightharpoonup{p_i^M} M^i. \tag{2}$$

for all $i \in \{1, \ldots, n\}$. Since all artifacts under consideration (models and metamodels) conform to the base $\mathbb{B}$, see sect. 5.1, commonalities among models $A^1 \in \mathbf{Mod}(M^1), \ldots, A^n \in \mathbf{Mod}(M^n)$ can be encoded in the same way, i.e. there is a graph $A^0$ of commonality representatives together with partial projections

$$A^0 \xrightharpoonup{p_i^A} A^i. \tag{3}$$

for all $i \in \{1, \ldots, n\}$. Again they can be specified in the same language as in listing 1.2, and can be stored physically, given that the modelling technique offers means to identify elements, e.g. primary keys in a database, position in an XML document, Uniform Resource Identificators (URIs) [5], etc.

The alignment of models $A^1$, $A^2$, and $A^3$ together with their commonalities is shown in fig. 2. Each cyan line represents a commonality representative and each line ends at the value under the respective projection. Some of the lines are binary, some ternary. In general, we would expect any arity, especially when the number $n$ of model spaces increases. The complete contents of fig. 2 is called a *comprehensive system*: the cyan connections its *commonalities* and models $A^1, \ldots, A^n$ its *components*.

Models $A^i$ are typed over their metamodels, i.e. there are typing morphisms $t_i : A^i \to M^i$ which can be combined to one big typing of all components. This typing extends to $A^0$ as well because elements $a_j$ and $a_k$ ($j \neq k$) of model components $A^j$ and $A^k$ are relatable only if their types $t_j(a_j)$ and $t_k(a_k)$ are related via a representative $w \in M^0$. Hence, a natural typing $t_0$ of a commonality representative $v$ of $a_j$ and $a_k$ is $t_0(v) := w$, such that

$$p_j^M(t_0(v)) = p_j^M(w) = t_j(a_j) = t_j(p_j^A(v)), \tag{4}$$

which shows that the typing extension $t_0$ integrates smoothly (respecting commonalities) into a typing of all parts of the comprehensive model, such that we end up with a *single typed* comprehensive system: $t : A \to M$.

#### 5.4 Reusing Methods of Local Model Management

Consider the OCL example and its generalization in terms of diagrammatic constraints in sect. 5.1. Theorem 1 in sect. 6 will show that comprehensive systems constitute a category basically with the same properties as the base language $\mathbb{B}$. Especially, pullbacks can be computed in a similar way, see Corollary 1 in sect. 6. Thus, we can define the consistency rules CR1-CR4 from sect. 3 as diagrammatic constraints $(\phi_i)_{i\in\{1,\ldots,4\}}$, now imposed on the comprehensive metamodel, which treat the commonality witnesses and projections as regular nodes and edges. Local constraints can be encoded as global constraints as well [24], such that we obtain comprehensive system $M_\Phi$ with a set $\Phi$ of constraints spanning local model elements but also elements of the linguistic extension. Any typed system $t : A \to M$ can then be checked against a constraint $\phi$ imposed via scope $d : S_\phi \to M$ by pullback of $d$ and $t$ in the category of comprehensive systems, see Theorem 1 in sect. 6. Hence, *query* implementation by pullbacks carries over from local models to comprehensive systems and we can reuse the theory of *diagrammatic constraints* to verify global consistency, which e.g. can be implemented by a straightforward translation of a respective model fragment and constraint to *Alloy* [20]. This can be used to formally verify that Fig. 2 is consistent w.r.t. CR1-CR4.

#### 5.5 Advantages over Model Merge

A merged model is an artifact which is computed *additionally* from local models $A^i$. Basically, it is the union of all elements of the $A^i$'s modulo their commonalities, see fig. 1. E.g. in the merge of models $A^1, A^2, A^3$ in fig. 2 there remains a single node, say `Diag/descr` of type `Var` (a type in $M^0$, see fig. 5), which represents sameness of `Diagnosis` $\in A^1$, `description` $\in A^2$ and `diagnosis` $\in A^3$.

We could implement global consistency rules on the merge by including the merge computation in the *check*-function as described in the algorithm in [24]. However, this leads to problems if the verification of a global constraint depends on the knowledge of containment in local models. This can be seen with consistency rule CR3 which relies om the containment of elements (in this case containment in $A^2$ and $A^3$). After merging `Diagnosis` and `description` into the single node `Diag/descr`, distinguishing its original local model would no longer be possible. In contrast, we do not loose this differentiation in comprehensive systems and can successfully check the validity of this constraint.

### 6 Categorical Formalization

This section is devoted to the formalization of comprehensive systems from sect. 5. In order to relate comprehensive systems to the TGG framework we need to employ *category theory (CT)* because TGGs are usually formulated in terms of CT. We recall the central terminology in the following section and refer to the introductory textbooks [4,34,50] for further references about CT.

#### 6.1 Theoretical Background and Notation

A *category* $\mathbb{C}$ is a collection of mathematical *objects* and of *morphisms*, which are means to compare objects. For a category $\mathbb{C}$, the set of objects is denoted $|\mathbb{C}|$ and for each pair $A, B \in |\mathbb{C}|$ the (hom-)set of morphisms from $A$ to $B$ is denoted by $Arr_{\mathbb{C}}(A, B)$. For each object $A \in |\mathbb{C}|$ there exists a special *identity* morphism $id_A : A \to A$. Moreover there is a neutral and associative *composition* operation $\circ : Arr_{\mathbb{C}}(A, B) \times Arr_{\mathbb{C}}(B, C) \to Arr_{\mathbb{C}}(A, C)$ for all $A, B, C \in |\mathbb{C}|$. The most prominent example is the base language of mathematics: $\mathbb{S}et$, the category of sets and total mappings. A category $\mathbb{C}$ is said to be *small*, if $|\mathbb{C}|$ is itself a set. *Equivalence* of two categories $\mathbb{C}$ and $\mathbb{D}$, written $\mathbb{C} \cong \mathbb{D}$, means that the network of objects and morphisms in $\mathbb{C}$ is identical to the one in $\mathbb{D}$ up to isomorphisms (e.g. bijections in $\mathbb{S}et$) between objects.

A *functor* provides the means to compare two categories $\mathbb{C}$ and $\mathbb{D}$: It is denoted $\mathbf{F} : \mathbb{C} \to \mathbb{D}$ and maps objects of $\mathbb{C}$ to objects of $\mathbb{D}$ and morphisms of each set $Arr_{\mathbb{C}}(A, B)$ to $Arr_{\mathbb{D}}(\mathbf{F}(A), \mathbf{F}(B))$. Moreover, it preserves identities and composition. $\mathbf{F}$ is called an *embedding*, if it is injective on objects of $\mathbb{C}$ and injective on $Arr_{\mathbb{C}}(A, B)$ for all $A, B \in |\mathbb{C}|$. For fixed categories $\mathbb{C}$ and $\mathbb{D}$ and functors $\mathbf{F}, \mathbf{F}' : \mathbb{C} \to \mathbb{D}$, a *natural transformation* $n : \mathbf{F} \Rrightarrow \mathbf{F}'$ is a family $(n_A : \mathbf{F}(A) \to \mathbf{F}'(A))_{A \in |\mathbb{C}|}$ of $\mathbb{D}$-morphisms compatible with images of $\mathbf{F}$ and $\mathbf{F}'$, i.e. for all $\mathbb{C}$-arrows $f : A \to B$: $n_B \circ \mathbf{F}(f) = \mathbf{F}'(f) \circ n_A$. In such a way we get a new category, the *functor category* $\mathbb{D}^{\mathbb{C}}$ with objects all functors from $\mathbb{C}$ to $\mathbb{D}$ and arrows the natural transformations. Functors $\mathbf{F} : \mathbb{C} \to \mathbb{S}et$ where $\mathbb{C}$ is small play a special role: $\mathbf{F}$ assigns to each $S \in |\mathbb{C}|$ a (*carrier*) *set* $\mathbf{F}(S)$ and for every $op \in Arr_{\mathbb{C}}(S, S')$ a mapping $\mathbf{F}(op) : \mathbf{F}(S) \to \mathbf{F}(S')$, i.e. $\mathbb{C}$ is a signature (think metamodel) that is interpreted by $\mathbf{F}$ (think instantiated). Hence, this is also called *functorial* or *indexed semantics* and $\mathbb{S}et^{\mathbb{C}}$ corresponds to the class of algebras for a signature $\mathbb{C}$ (instance worlds for a metamodel). E.g. objects of $\mathbb{G} := \mathbb{S}et^{\mathbb{B}}$ are E-Graphs, if $\mathbb{B}$ is the category depicted in fig. 4b (identities are omitted) and E-Graph-homomorphisms are exactly the natural transformations. For set-based structures, we use the notation $A \hookrightarrow B$ to indicate included structures ($A$ in $B$) such as subsets or subgraphs.

*Universal constructions* in categories have proven to be of importance in many software theoretical methods. Intuitively universal constructions can be described as a generalization of *meets* and *joins* in a preorder. Some well known examples for universal constructions in $\mathbb{S}et$ are cartesian products or disjoint unions (coproduct). It is important to note that $\mathbb{S}et$ possesses all these universal constructions and thus every category $\mathbb{S}et^{\mathbb{C}}$ does as well, where the computation of universal constructions is carried out "pointwise".

#### 6.2 Comprehensive System

We begin the formalization of comprehensive systems by fixing a sufficiently large natural number $n$ and considering a synchronization scenario with model spaces $(\mathbf{Mod}(M^j_{\Phi_j}))_{j\in\{1,\ldots,n\}}$. E.g. UML class diagrams, BPMN process models and DMN tables.

**Definition 1 (Base Modelling Language).** *The base modelling language is a small category* $\mathbb{B}$.

In order to distinguish between the different system components, we will work with copies $\mathbb{B}_j$ of $\mathbb{B}$. We let $|\mathbb{B}_j| = \{s_j \mid s \in |\mathbb{B}|\}$ and similarly $op_j : s_j \to s'_j$ be an arrow in $Arr_{\mathbb{B}_j}$, if $op : s \to s'$ is an arrow of $Arr_{\mathbb{B}}$.[1]

**Definition 2 (Comprehensive Systems, Components, Commonalities).** *A* comprehensive system $C$ *consists of*

- *Functors* $C_j : \mathbb{B}_j \to \mathbb{S}et$ *for each* $j \in \{1,\ldots,n\}$, *called* Components
- *A functor* $C_0 : \mathbb{B}_0 \to \mathbb{S}et$ *determining the* Commonality representatives, *and*
- *A collection of* partial *functions* $(C_0(s) \overset{p_{j,s}}{\rightharpoonup} C_j(s))_{s\in|\mathbb{B}|,1\le j\le n}$, *called* projections, *establishing the* commonalities *of* $C$,

*such that for all* $op : s \to s' \in \mathbb{B}$ *and* $1 \le j \le n$ *the following statement holds:*

$$\text{If } p_{j,s}(x) \text{ is defined, then } p_{j,s'}(C_0(op_0)(x)) \text{ is defined} \tag{5}$$
$$\text{and } p_{j,s'}(C_0(op_0)(x)) = C_j(op_j)(p_{j,s}(x)). \tag{6}$$

Note that (5) and (6) generalize the edge-node-incidences, see sect. 5.2, which we already semi-formalized in (1). In the sequel, the index of functors $C_i$ will be omitted, since it can be derived from the domain of definition. Hence, a comprehensive system is a *single* functor $C$ with domain the $n+1$ copies of $\mathbb{B}$ and $(n+1)b$ carrier sets, if $b$ is the cardinality of $|\mathbb{B}|$: In view of the introductory remarks on functors in sect. 6.1, $C_0,\ldots,C_n$ can be seen as $n+1$ instance worlds for metamodel $\mathbb{B}$, e.g. E-Graphs, each with $b = 4$ carrier sets.

The fundamental *linguistic extension* are the partial functions. They act according to our example in sect. 5.2: In the tuple $(p_1(w),\ldots,p_n(w))$ the $p_j$ determine sameness of its components based on representative $w$.

[1] The abbreviation "op" for arrows of the base shall indicate that $\mathbb{B}$-arrows are certain operations constituting the structure of the base language, such as source and target operations of edges in graphs.

The next definition deals with different comprehensive systems. In this case, it is necessary to tell the respective partial mappings apart, such that we write $p^C_{j,s}$, if we depict the mappings in the particular system $C$.

**Definition 3 (Homomorphisms between Comprehensive Systems).** *Let $C, C'$ be comprehensive systems as defined in Def. 2. A* homomorphism between comprehensive systems *is a family*

$$(f_{i,s} : C(s_i) \to C'(s_i))_{s \in |\mathbb{B}|, 0 \leq i \leq n}$$

*of mappings compatible with arrows, i.e. $\forall i \in \{0, \ldots, n\}, \forall op : s \to s' \in Arr_{\mathbb{B}}$: $f \circ C(op_i) = C'(op_i) \circ f$, and compatible with partial mappings: For all $j \in \{1, \ldots, n\}$, $s \in |\mathbb{B}|$ and $x \in C(s_0)$:*

$$\text{If } p^C_{j,s}(x) \text{ is defined, then } p^{C'}_{j,s}(f(x)) \text{ is defined and } p^{C'}_{j,s}(f(x)) = f(p^C_{j,s}(x)) \quad (7)$$

*where we write $f$ instead of $f_{j,s}$, if the indexing becomes clear from the context.*

A typical example is a typing morphism $t : A \to M$ for two comprehensive systems $A$ and $M$. Then equation (7) reflects property (4), i.e. compatibility of commonalities and typing. This can be seen in fig. 2: The complete contents of it is a comprehensive system $A$ typed over the comprehensive metamodel $M$ partly depicted in fig. 5. $A^0$ consists of all cyan (binary or ternary) lines and $p_{j,s}$ assigns to a line its line end in model $A^j$, where $s$ is the respective element type (node or edge).

**Proposition 1.** *Comprehensive Systems together with homomorphisms between them constitute a category $\mathbb{CS}$.*

*Proof.* An identity is a family of identities, composition is composition of mappings $f_{j,s}$. This yields neutrality and associativity. Moreover, composed homomorphisms are still compatible with arrows. Whereas this follows in the usual way for $op : s \to s'$, transitivity of the definedness implication in (7) also yields compatibility with partial functions. □

#### 6.3 Multimodel Equivalence

An alternative but closely related approach to our construction is to consider commonalities, i.e. commonality representatives $A^0$ together with projections $(p^A_j)_{1 \leq j \leq n}$, not represented *internally* by means of the modelling technique but *externally* as $n$ spans of morphisms [24,46]. Let for this $\mathbb{G} := \mathbb{S}et^{\mathbb{B}}$, see the remarks on functor categories in sect. 6.1. The resulting artifacts of the category in [46] is a subcategory $\mathbb{M}$ of the functor category $\mathbb{G}^{\mathbb{I}}$, where $\mathbb{I}$ is defined as in fig. 6 (identity arrows of $\mathbb{I}$ are again omitted). It is a subcategory, because it only consists of those functors $\mathbf{M} : \mathbb{I} \to \mathbb{G}$, for which the images $\mathbf{M}(\overline{-j})$ of the top arrows in fig. 6 are monic (i.e. are monomorphisms).

The proof of the following theorem relies mainly on cartesian closedness of the category of small categories, i.e. $\mathbb{G}^{\mathbb{I}} \cong \mathbb{S}et^{\mathbb{B}\times\mathbb{I}}$ (internalization) and the fact that spans with one monic leg represent partial mappings, the middle object of the span being the domain of definition of the partial map. A detailed proof of the theorem is given in [47].

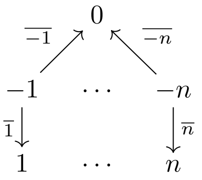

**Fig. 6.** Category $\mathbb{I}$

**Theorem 1 (Equivalence of Categories).** $\mathbb{CS} \cong \mathbb{M}$.

**Corollary 1.** $\mathbb{CS}$ *possesses all pullbacks and they are computed separately for the commonality representatives and for each component.*

*Proof.* Follows from Theorem 1 and the fact that functor categories possess all pullbacks, their pointwise construction guaranteeing that spans with one monic leg are preserved, because pullbacks preserve monomorphisms. □

Auxiliary commonality structures have been used for model synchronization in the TGG framework [40]: Consistency relations between two model spaces are defined declaratively by a grammar. The grammar rules are defined over triple graphs, i.e. pairs of graphs connected by special *correspondence*-graphs, which resemble structural commonalities. From the grammar rules, procedures for consistency verification [27], model transformation [13] and (concurrent) model synchronization [19,18] can automatically be derived. The solution space, however, is limited to binary scenarios. Trollmann and Albayrak [48,49] generalized the TGG framework to cope with multiple models within a *graph diagram* (GD) framework. If we assume that the involved models are also objects of the graph-like category $\mathbb{G}$ (see above), then graph diagrams are the objects of a functor category $\mathbb{G}^{\mathbb{X}}$, but with a different schema category $\mathbb{X}$: It has objects $|\mathbb{X}| = R \sqcup N$ and all non-identity morphisms connect a source from $R$ (relations) to a target from $N$ (models). There is at most one arrow in $Arr_{\mathbb{X}}(r, m)$ for fixed $r \in R$ and $m \in N$. In such a way graph diagrams, i.e. functors $\mathbf{D} : \mathbb{X} \to \mathbb{G}$ can specify relations of different arities.

They are, however, *static*: If $r \in R$ has $k$ outgoing morphisms with targets $m_1, ..., m_k \in N$, $\mathbf{D}(r)$ is a $k$-ary correspondence relation with representatives which relate exactly one element in each of the $k$ models $\mathbf{D}(m_j)$. Consequently, the schema category has to change each time a new relation is added!

Graph diagrams (GD) subsume TGGs, which have schema $\mathbb{X}_{TGG} := 1 \xleftarrow{s} 0 \xrightarrow{t} 2$, i.e. $R = \{0\}$ and $N = \{1, 2\}$. Computations of triple graphs (and graph diagrams) during rule application as well as decomposing GD rules for forward and backward transformations are based on *pushout* constructions in $\mathbb{G}^{\mathbb{X}}$. In the rest of the section we show that our framework is more general than graph diagrams in that there is an embedding functor $\mathbf{T} : \mathbb{G}^{\mathbb{X}} \to \mathbb{CS}$, the *translation functor*, which preserves pushouts and hence is able to replay all GD computations in our framework, yet being able to cope with new relations *without* changing the schema category.

We use the following notations: For a morphism $f : A \to B$ in a category $\mathbb{C}$ we write $A = \mathrm{dom}(f)$ and $B = \mathrm{codom}(f)$ for its domain and codomain and we use the shorthand notation $Arr_{\mathbb{C}}(\_, B) := \{f \in Arr_{\mathbb{C}} \mid codom(f) = B\}$. We write $\coprod_{i \in I} D_i$ to depict the coproduct of a collection $(D_i)_{i \in I}$ of $\mathbb{G}$-objects. Note that a collection $(D_i \xrightarrow{f_i} D)_{i \in I}$ of morphisms yields the morphism $\coprod_{i \in I} f_i : \coprod_{i \in I} D_i \to D$ by the universal property of coproducts, i.e. the morphism, which acts as $f_i$ on each $D_i$.

By Theorem 1, it suffices to define a functor from $\mathbb{G}^{\mathbb{X}}$ to $\mathbb{M}$. The composition of this functor with the equivalence will yield the desired result. This functor will also be called $\mathbf{T}$. Let a schema category $\mathbb{X}$ for graph diagrams be given with $|\mathbb{X}| = R \uplus N$ and let $n$ be the cardinality of $N$. Without loss of generality, we assume $N = \{1, \ldots, n\}$. Let $\mathbf{D}$ be a graph diagram, then we define a multimodel $M := \mathbf{T}(\mathbf{D})$ intuitively as follows (recall the multimodel schema in fig. 6): The model components of $N$ are the same as those of $\mathbf{D}$, the commonality specification $M(0)$ is the disjoint union of all relations in $\mathbf{D}$, the middle objects $M(-j)$ are the union of those relations, the model $\mathbf{D}(j)$ participates in:

$$
\begin{array}{ll}
M(j) := \mathbf{D}(j) & \text{(Models are untouched)} \\
M(0) := \coprod_{r \in R} \mathbf{D}(r) & \text{(Coproduct of all relations)} \\
M(-j) := \coprod_{f \in Arr_{\mathbb{X}}(\_, j)} \mathbf{D}(\mathrm{dom}(f)) & \text{(Participating Relations of } \mathbf{D}(j))
\end{array}
$$

for all $j \in \{1, \ldots, n\}$. Furthermore,

$$
\begin{array}{ll}
M(\bar{j}) = \coprod_{f \in Arr_{\mathbb{X}}(\_, j)} \mathbf{D}(f) & \text{(Projections)} \\
M(\overline{-j}) : \coprod_{f \in Arr_{\mathbb{X}}(\_, j)} \mathbf{D}(\mathrm{dom}(f)) \hookrightarrow \coprod_{r \in R} \mathbf{D}(r) & \text{(Domains)}
\end{array}
$$

Hence projections $M(\bar{j})$ are the unions of the domains of those relating morphisms that have target $\mathbf{D}(j)$ and inclusions arise from the fact that coproducts in the above definition of $M(-j)$ (taken over some relations) are always subgraphs of the complete coproduct $M(0)$ (which is taken over all relations).

The definition of $\mathbf{T}$ on arrows is straightforward and we give it only informally: If $n : \mathbf{D} \Rrightarrow \mathbf{D}'$ is an arrow between graph diagrams, then (1) $\mathbf{T}(n)_i$ is a morphism which acts in the same way as $n_i$ on $\mathbf{D}(i)$, if $i > 0$, (2) it amalgamates the actions of $n$ on relations, if $i = 0$, which (3) naturally restricts to the respective actions, if $i < 0$. It is then easy to see, that $\nu := \mathbf{T}(n)$ is again a natural transformation.

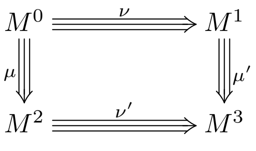

**Fig. 7.** Pushout in $\mathbb{M}$

**Theorem 2.** *Functor* $\mathbf{T} : \mathbb{G}^{\mathbb{X}} \to \mathbb{CS}$ *is an embedding and preserves pushouts.*

For a detailed proof of this theorem consult [47]. To sketch the idea, note that we cannot rely on pointwise pushout construction alone: Given a span $(\nu, \mu)$ in $\mathbb{M}$ as in fig. 7, pointwise pushout construction may fail to belong to $\mathbb{M}$! E.g. if $\nu$ and $\mu$ are arbitrarily given, then $M^3$ in fig. 7 may not be admissible for $\mathbb{M}$ because the mapping $M^3(\overline{-j})$ may fail to be monic, an effect already studied in [25, Example 6]

Instead the proof uses the fact that naturality squares in $\nu$ are pullbacks, if $\nu$ is in the image of $\mathbf{T}$. Then *hereditariness* [17] of pushouts in $\mathbb{G}$ yields admissibility of $M^3$ and nevertheless allows for pointwise pushout construction. We obtain as a consequence:

**Corollary 2.** *Every sequence of rule applications in* $\mathbb{G}^{\mathbb{X}}$ *has a unique representation of corresponding rule applications in* $\mathbb{CS}$ *and hence can be replayed in the general framework of comprehensive systems.* □

### 7 Conclusion, Related Work and Future Plans

Our work can be summarized by the slogan "from many models to one model": Multimodelling is addressed by a construction that yields a single artifact, where existing means for consistency verification and restoration can be reused. Over many years such global artifacts were computed via merging [38,6,36,10], which poses several difficulties especially if the verification of a global constraint depends on the knowledge of which local model the elements came from. Hence, we proposed comprehensive systems that mitigate issues with the former and represent a generalization of graph diagrams and triple graphs—alternatives to our approach. Comprehensive systems stress the utility of *partial* mappings in commonality specifications, which have been promoted in [46] and were also picked up in [25].

Related work on multimodel consistency management was surveyed in sect. 4. Thus, at this point we mainly want to place our contribution in this landscape. Our approach can be considered as a *structural* one and is in tradition with other approaches based on *traceability links*. Recent other representatives in this line are [16], which uses binary links to relate different artifacts in a practical scenario, and [21], which develops a language, similar to ours, for expressing commonalities for global consistency restoration. All these works share the requirement for a common meta-metalanguage: In our case, given by graph-like structures (presheaf topoi). A rather different approach is the framework proposed by Stevens [45]: It considers consistency restoration to be performed locally by a builder. The concrete implementation of the builder is up to the user and thus there is no requirement for a common meta-metalanguage. The global coordination of multiple builder is handled by the framework, controlled by an orientation model. Comparing Stevens approach to structural approaches, the former is more abstract and thus allows more directions for tooling implementation, whereas structural approaches allow formal analysis of the nature of consistency rules. It will be worthwile to investigate the relationship between both approaches in the future.

This paper provides the framework for performing multi model consistency management by reusing existing restoration techniques. We plan to address the momentary lack of practical evidence by investigating *model repair* [28] as the next step. Being conceptually close to TGGs, grammar-based approaches seem a natural fit but we plan to experiment with solver-based approaches as well, further taking into account: Human interaction and learning.
